# Physical Activity Is Associated With Sleep Quality: Results of the ESSE-RF Epidemiological Study

**DOI:** 10.3389/fpsyg.2021.705212

**Published:** 2021-08-05

**Authors:** Elena Dubinina, Lyudmila S. Korostovtseva, Oxana Rotar, Valeria Amelina, Maria Boyarinova, Mikhail Bochkarev, Tatiana Shashkova, Elena Baranova, Roman Libis, Dmitry Duplyakov, Yurii Sviryaev, Aleksandra Konradi, Eugene Shlyakhto

**Affiliations:** ^1^Clinical Psychology Department, Herzen State Pedagogical University of Russia, Saint Petersburg, Russia; ^2^Laboratory of Clinical Psychology and Psychodiagnostics, V.M. Bekhterev National Research Medical Center for Psychiatry and Neurology, Saint Petersburg, Russia; ^3^Almazov National Medical Research Centre, Saint Petersburg, Russia; ^4^Department of Health of Khanty-Mansi Autonomous Region Yugra, Khanty-Mansiysk State Medical Academy, Khanty-Mansi, Russia; ^5^Department of Internal Diseases #2 with the course of endocrinology, cardiology and functional diagnostics n.a. G.F. Lang with the in-patient clinic, Pavlov First Saint Petersburg State Medical University, Saint Petersburg, Russia; ^6^Department of Internal Diseases, Orenburg State Pedagogical University, Orenburg, Russia; ^7^Department of Cardiology and Cardiosurgery, Samara State Medical University, Samara, Russia; ^8^Institute of Translational Medicine, ITMO University, Saint Petersburg, Russia

**Keywords:** sleep quality, physical activity, anxiety, depression, socioeconomic factor, sleep duration, insomnia, sleep disorders

## Abstract

**Background and hypothesis:**

Physical activity (PA) is an important behavioral factor associated with the quality of life and healthy longevity. We hypothesize that extremely low and extremely high levels of daily PA (including occupational PA) may have a negative impact on sleep quality and psychological well-being.

**Objective:**

The aim of the study is to investigate the association between the level and type of PA and sleep problems in adult population.

**Materials and methods:**

The sample of the study consisted of the participants from the population-based cohort of The Epidemiology of Cardiovascular Risk Factors and Diseases in Regions of the Russian Federation Study (ESSE-RF). The data of three regions (Saint Petersburg, Samara, Orenburg), varying in geographic, climatic, socioeconomic characteristics, was included into analysis. The total sample consisted of 4,800 participants (1,600 from each region; 1,926 males, 2,874 females), aged 25–64. The level of PA was evaluated using three parameters: the type of PA at work, the frequency of an intensive/high PA including sport (times a week), the mean duration of leisure-time walking (minutes a day). The measures of sleep quality were sleep duration and the frequency of difficulty falling asleep, difficulty maintaining sleep, daytime sleepiness, and sleep medication use. PA and sleep characteristics were assessed by interview carried by the trained medical staff.

**Results:**

When controlling for gender, age and socioeconomic status (SES) extremely high occupational PA was a significant risk factor for difficulty falling asleep three or more times a week [OR(CI95%) = 1.9(1.2–3.0), *p* = 0.003] while working in a sitting position or having moderate physical load at work were not associated with sleep characteristics. Having a high physical load six or more times a week was a risk factor for difficulty falling asleep controlling for gender, age and SES [OR(CI95%) = 1.9(1.4–3.4), *p* = 0.001]. The association between leisure-time walking and sleep characteristics was insignificant. Walking less than an hour a day was associated with increased depression scores (46.5 vs. 41.9%, *p* = 0.006).

**Conclusion:**

High physical load at work and excessively frequent intensive PA are associated with difficulties initiating sleep and may represent a risk factor for insomnia.

## Introduction

Physical activity (PA), sleep, dietary pattern, and psychological well-being are known to be important factors contributing to the quality of life and healthy longevity. Interrelations between these components and other factors potentially influencing quality of life are being extensively studied.

According to a recent systematic review (Cunningham et al., 2020), PA in older adults (≥60 years) reduces risk of all-cause mortality, cognitive decline, and depression. Participants with sleep disorders improved sleep quality as a result of increased weekly activity up to 100 (Reid et al., 2010)—210 min (King et al., 2008) on a regular basis—from 16 (King et al., 1997) to 54 weeks (King et al., 2008).

In a systematic review by Yang et al. (2012) middle-aged and older adults with sleep problems who practiced physical exercise program (10–16 weeks) which consisted of either moderate intensity aerobic exercise or high intensity resistance exercise showed significantly reduced sleep latency (assessed by Pittsburgh Sleep Quality Index score) and medication use compared to the control group. In a more recent systematic review covering 14 studies all but one study found at least one significant improvement on sleep outcomes in generally healthy older adults engaged in PA programs, and no significantly detrimental effects were reported (Vanderlinden et al., 2020). The authors conclude that physical exercise may be regarded an alternative or complementary approach to existing interventions for sleep disorders.

The agreement on the optimal level of physical load beneficial for sleep quality and general physical functioning is being established. WHO recommends 150 min moderate-intensity exercise weekly World Health Organisation (WHO) (2010). According to Hartescu et al. (2015), this level of exercise is associated with better sleep and mood. Authors emphasize that these results were independent of participants’ social status, health and daily light exposure. Vancampfort et al. (2018) examined the relationship between compliance with the PA recommendations of the World Health Organization (150 min of moderate to vigorous PA per week for prevention or reducing sleep problems) in 38 low- and middle-income countries (*n* = 204,315). After adjusting for socio-demographic characteristics, obesity, chronic physical diseases, depression, and anxiety, not complying with WHO recommendations was associated with 1.23 higher risk for sleep problems.

It is supposed that relationship between PA and sleep is bidirectional and poor sleep may also contribute to reduced PA (Kline, 2014; Dolezal et al., 2017), making a vicious circle that maintains reduced quality of life.

Studies revealed possible pathways for PA effects on sleep involving endocrine, autonomic nervous system, metabolism, circadian rhythm, and somatic functions (Uchida et al., 2012).

On the whole studies confirm beneficial effects of PA on sleep quality but, as recent studies demonstrated, this relationship is more complex than a linear association. Thus there are some controversies concerning the role of type of PA, its regimen, and intensity.

There is evidence that vigorous PA (compared to moderate PA) does not have any positive impact on sleep quality (Myllymäki et al., 2011; Pengpid and Peltzer, 2018).

According to a cross-national study, including 9,238 adults from five countries, both very low (<10 continuous min per week) and very high (>300 min per week) levels of regular physical activity are associated with the risk of insomnia (Hartescu and Morgan, 2019).

Additionally when the type of PA is considered, high occupational PA (compared to leisure-time PA) may have detrimental effects on physical and mental health, including sleep (Soltani et al., 2012; Wennman et al., 2014; Skarpsno et al., 2018a; Cillekens et al., 2020). Skarpsno et al. (2018b) studied the association of heavy physical work resulting in fatigue with risk of sleep disorders and chronic pain as a potential mediator of this association. Authors conclude that there is a link between work-related physical fatigue and musculoskeletal pain that should be considered as a target for prevention of insomnia in working populations. It can also be assumed that exposure to stress is another factor involved in an interplay between heavy physical work and sleep disturbances. Thus, the contribution of different levels and types PA to sleep quality still needs to be elucidated. It is exceptionally relevant due to high comorbidity between sleep disturbances and mental disorders. Results of epidemiological study indicate that 40% of patients with insomnia have comorbid psychiatric disorder. Major depressive disorder or dysthymia is detected in 23%, anxiety disorders in 24% (Ford and Kamerow, 1989). In a meta-analysis, covering 21 longitudinal epidemiological studies, non-depressed persons with insomnia had a twofold risk of developing depression, compared to persons who had no sleep problems (Baglioni et al., 2011). It is shown that treatment of a psychiatric disorder can lead to improvement in sleep quality, and treatment of sleep disorders can have a beneficial effect on the course of psychiatric disorder (Krystal, 2012).

Thus, it is essential for the assessment of groups at high risk of developing sleep and mental disorders and in order to plan preventive activities to understand the nature of interaction between such factors involved in quality of life as sleep, PA, and psychological well-being.

Objective of the present study is to investigate the association between the level and type of physical activity (PA) and sleep problems in adult population taking into account psychological well-being as possible mediating factor.

We hypothesize that extremely low and extremely high levels of daily PA (including occupational PA) may have a negative impact on sleep quality and psychological well-being.

The results of the study may contribute to developing recommendations for optimal level of leisure and occupational PA to prevent sleep disorders, anxiety and depression.

## Materials and Methods

### Sample

The sample of the study consisted of the participants from the population-based cohort of The Epidemiology of Cardiovascular Risk Factors and Diseases in Regions of the Russian Federation Study (ESSE-RF) that was conducted in 2012–2013 and covered 13 regions of Russian Federation (Boitsov et al., 2013). The protocol was approved by the Local Ethical Committee (Almazov National Medical Research Centre), approval #193 dated by 8 October 2012. All patients/participants provided written informed consent prior participation in the study.

The data of three regions (Saint-Petersburg, Samara, Orenburg), varying in geographic, climatic, socioeconomic characteristics, was included into analysis in our study.

The total sample consisted of 4,800 participants (1,600 from each region), aged 25–64 years.

Data on socio-demographic characteristics (age, gender, education, marital status, employment status, income level), PA and sleep quality were obtained in a structured interview that was carried out by trained medical staff in presence.

Weight and height were measured during the interview by medical staff, and the body mass index (BMI) was calculated using the Quetelet equation [weight (kg)/height^2^ (m^2^)]. Height was assessed with the accuracy up to 0.5 cm, in the standing position, by stadiometer (Medical RP, TVES, Russia), weight was measured with the accuracy up to 100 g by medical scales (HEM-150 MASSA-K, Russia).

The sample was divided in two age groups: young (25–44 years old) and middle-aged (45–64 years old). The threshold of 45 years was chosen based on WHO classification of age groups.

The main sample characteristics are represented in the Table 1.

**TABLE 1:** Sample characteristics.

**Sample characteristics**		***n***	**%**
Gender	Male	1,926	40.1
	Female	2,874	59.9
Age	young (25–44 years)	2,091	43.6
	middle-aged (45–64 years)	2,709	56.4
Education	Higher	2,354	49.0
Employment status	Working	4,009	83.5
	Unemployed	312	6.5
	Retired	479	10.0
Marital status	Married	3,081	64.2
	Single	822	17.1
	Divorced	602	12.5
	Widowed	295	6.1
Self-reported financial status	Wealthy	13	0.3
	Relatively wealthy	487	10.1
	Average	3,817	79.5
	Relatively poor	447	9.3
	Poor	36	0.8
Socioeconomic status (SES)^1^	Higher	2,177	45.4
	Lower	2,623	54.6
BMI	<18.5 kg/m^2^	92	1.9
	≥18.5, <25 kg/m^2^	1,630	34.0
	≥25, <30 kg/m^2^	1,812	37.8
	≥30 kg/m^2^	1,266	26.4

*^1^Higher SES is defined as a combination of higher education and middle and higher financial status.*

### Measures

The level of PA was evaluated during interview by asking several questions from commonly used validated questionnaires (Supplementary Table 1). The following parameters were assessed:

1. The type of PA at work (for working participants): (a) mainly sitting, (b) mainly walking, (c) lifting and carrying small loads, and (d) heavy physical work.
2. The frequency of an intensive/high PA including sport (times a week). In the case of self-reported disease as a barrier to PA the data were excluded from the analysis.
3. The mean duration of leisure-time walking (minutes a day).

Valid data on the level of PA at work were available for 4,696 participants, valid data on the frequency of high physical load per week were available for 2,796 participants, on leisure-time walking—for 4,084 participants. The data of 49 respondents (16 males, 33 females), who indicated that their physical disease was a barrier to a high PA, were excluded from analysis in relation to a high PA.

Sleep quality was assessed based on five questions regarding sleep duration and sleep problems (described in Korostovtseva et al., 2020) (Supplementary Table 1).

The measures of sleep quality were the following:

1. Sleep duration.
2. Difficulty falling asleep.
3. Difficulty maintaining sleep.
4. Daytime sleepiness.
5. Sleep medication use.

In accordance with the European guideline for the diagnosis and treatment of insomnia (Riemann et al., 2017) difficulty falling asleep, maintaining sleep, uncontrolled daytime falling asleep three and more times a week were considered as sleep problems.

The other signs of disturbed sleep and unhealthy sleep behavior were sleep duration less than 6 h and using sleep medication at least once (Sivertsen et al., 2015; Watson et al., 2015).

Anxiety and depression were assessed using a Russian validated version of the Hospital Anxiety and Depression Scale (HADS) (Zigmond and Snaith, 1983; Smulevich, 2007). Cut-offs for increased level of anxiety and depression were eight points and higher.

### Statistical Analysis

Statistical analysis was made with IBM SPSS 21.0 software.

Assessment of differences between subgroups by quantitative variables (mean leisure-time walking, BMI) was carried out with Student’s *t*-test.

Chi-square test was applied to examine the association of qualitative and rank variables such as type of PA at work, frequency of high physical load (times per week), frequency of sleep problems (more or less than three times a week), increased or normal level of anxiety and depression.

The association of PA with sleep problems was examined by logistic regression analysis with forward (likelihood ratio) method of variable selection. Simple and multiple binary logistic models were used with the following dependent variables: sleep duration less than 6 h, experience of sleep medication use, difficulties falling asleep, staying asleep and episodes of daytime sleepiness three or more times a week. When an association of PA with sociodemographic characteristics was found these variables were included into the model as covariates. Results are presented as odds ratios (OR) with 95% confidence intervals (CIs). Nagelkerke R^2^ was calculated to assess the contribution of independent variables to the variation of dependent variables.

## Results

### The Level of PA

Among working participants 2,212 (55.2%) are mainly sitting at work, 1,304 (32.5%) are predominantly walking, 353 (8.8%) are lifting and carrying small loads, 140 (3.5%) perform heavy physical work.

Females [1,359 (59.5%) compared to 853 (49.4%) males *p* < 0.0001] and participants of the higher-SES group [1,326 (68.9%) compared to 886 (42.5%) in the lower-SES group, *p* < 0.0001] more frequently reported working primarily in a sitting position.

Among those, who did not indicate some disease as a barrier to a high PA, 735 (26.8%) participants have a high physical load (including sport and intensive physical exercise) less than one time a week, 1,050 (38.2%)—one or two times a week, 732 (26.6%) participants—from 3 to 5 times a week, 230 (8.4%)—six times a week or every day.

In the whole group 391 (9.6%) participants have leisure-time walking less than 30 min a day, 1,378 (33.7%) participants are walking from 30 min to an hour a day, 1,257 (30.8%)—from one to 2 h, 1,058 (25.9%)—more than 2 h a day.

Participants of the older (45–64) age group [76.3 ± 1.1 compared to 68.4 ± 1.1 min in the younger age group (25–44), *t* = −5.07, *p* < 0.0001], females (74.4 ± 1.0 compared to 70.6 ± 1.2 min in males, *t* = −2.38, *p* < 0.05) and individuals with lower SES (78.4 ± 1.1 compared to 66.6 ± 1.1 min in the higher-SES group, *t* = 7.64, *p* < 0.0001) spend more leisure-time walking.

The data on PA in the sample are similar to the Russian national averages estimated in ESSE-RF epidemiological study for 13 regions (Balanova et al., 2014).

The characteristics of PA were not associated with BMI, but middle-aged persons (compared to younger subgroup) (28.78 ± 0.10 vs. 25.28 ± 0.10, *t* = −24.38, *p* < 0.0001) and persons with lower SES (compared to higher SES) (27.94 ± 0.10 vs. 26.41 ± 0.11, *t* = 10.21, *p* < 0.0001) had higher BMI.

### PA and Sleep Characteristics

The frequency of sleep complaints was assessed in individuals with different level of PA (Table 2). Differences between subgroups were calculated using Chi-square and Student’s *t* tests. Most salient differences concern difficulty falling asleep. This sleep problem is significantly more frequent in participants who regularly or constantly have a very high physical load. Also persons with difficulties initiating sleep as well as staying asleep spend more time walking, probably as a way to cope insomnia. In addition, more frequent sleep medication intake was found in individuals with more frequent high physical load.

**TABLE 2:** Physical activity (PA) and sleep characteristics.

**Characteristics of PA**		**Mean sleep duration**		**Difficulty falling asleep**		**Difficulty staying asleep**		**Sleepiness: episodes of daytime falling asleep**		**Sleep medication use**	
		**<6 h**	**≥6 h**	**≥3 times a week**	**no or <3 times a week**	**≥3 times a week**	**no or <3 times a week**	**≥3 times a week**	**no or<3 times a week**	**At least once**	**Never**
Type of PA at work, *n* (%)	sitting	96 (4.3)	2,116 (95.7)	231 (10.4)	1,981 (89.6)	170 (7.7)	2,042 (92.3)	94 (4.2)	2,118 (95.8)	306 (13.8)	1,906 (86.2)
	walking	48 (3.7)	1,256 (96.3)	140 (10.7)	1,164 (89.3)	110 (8.4)	1,194 (91.6)	54 (4.1)	1,250 (95.9)	182 (14.0)	1,122 (86.0)
	carrying loads	23 (6.5)	330 (93.5)	57 (16.1)	296 (83.9)	39 (11.0)	314 (89.0)	24 (6.8)	329 (93.2)	66 (18.7)	287 (81.3)
	heavy physical work	8 (5.7)	132 (94.3)	27 (19.3)	113 (80.7)	14 (10.0)	126 (90.0)	5 (3.6)	135 (96.4)	23 (16.4)	117 (83.6)
Chi-square/p-level		5.99/0.112		19.13/ < 0.0001		5.16/0.16		5.36/0.15		6.54/0.09	
Frequency of high physical load, *n* (%)	<1 time a week	43 (5.9)	692 (94.1)	125 (17.0)	610 (83.0)	74 (10.1)	661 (89.9)	35 (4.8)	700 (95.2)	117 (15.9)	618 (84.1)
	1–2 times a week	38 (3.6)	1,012 (96.4)	143 (13.6)	907 (86.4)	110 (10.5)	940 (89.5)	47 (4.5)	1,003 (95.5)	154 (14.7)	896 (85.3)
	3–5 times a week	37 (5.1)	695 (94.9)	100 (13.7)	632 (86.3)	75 (10.2)	657 (89.8)	42 (5.7)	690 (94.3)	146 (19.9)	586 (80.1)
	>5 times a week	8 (3.5)	222 (96.5)	52 (22.6)	178 (77.4)	33 (14.3)	197 (85.7)	18 (7.8)	212 (92.2)	43 (18.7)	187 (81.3)
Chi-square/p-level		5.94/0.12		14.95/0.002		3.73/0.29		5.07/0.17		9.62/0.02	
Mean leisure-time walking, min., M ± m		78.9 ± 4.0	72.6 ± 0.8	80.4 ± 2.3	71.7 ± 0.8	78.8 ± 2.5	72.2 ± 0.8	78.3 ± 4.1	72.6 ± 0.8	74.5 ± 2.0	72.6 ± 0.8
t/p-level		1.7/0.09		3.75/ < 0.001		2.59/0.01		1.52/0.13		0.89/0.37	

Logistic regression was applied to assess the contribution of PA characteristics to the risk of sleep disturbances.

Performing heavy physical work was found to be a risk factor for difficulty falling asleep three or more times a week [OR(CI95%) = 1.9(1.2–3.0), *p* = 0.003, *B* = 0.65, and *R*^2^ = 0.004], although its contribution is relatively small. When adjusted for gender, age and SES (Table 3) performing heavy physical work remained a statistically significant risk factor (*R*^2^ = 0.06) while working in a sitting position or having moderate physical load at work were not associated with sleep characteristics.

**TABLE 3:** Logistic regression model for prediction of difficulty falling asleep.

**Variable**	**B**	**OR**	**p-level**	**CI95%**
Heavy physical work	0.77	2.2	0.001	1.4–3.4
Female	0.55	1.7	<0.0001	1.4–2.2
Lower SES	0.36	1.4	0.001	1.2–1.8
Middle age	0.80	2.2	<0.0001	1.8–2.8

Having a high physical load six or more times a week was a risk factor for difficulty falling asleep [OR(CI95%) = 1.9(1.3–3.0), *p* = 0.003, *B* = 0.65, and *R*^2^ = 0.004). The contribution is small but statistically significant. And it remained statistically significant [OR(CI95%) = 1.9(1.4–3.4), *p* = 0.001, and *B* = 0.77] after controlling for gender [OR(CI95%) = 1.9(1.8–2.8), *p* < 0.0001, and *B* = 0.55], age [OR(CI95%) = 2.2(1.8–2.8), *p* < 0.0001, and *B* = 0.80] and SES [OR(CI95%) = 1.4(1.2–1.8), *p* < 0.0001, and *B* = 0.36]; *R*^2^ = 0.06.

No association was found between leisure-time walking and sleep characteristics.

### Physical Activity, Anxiety, and Depression

Characteristics of PA in the sample were also analyzed in relation to the level of anxiety and depression (HADS scores) (Table 4). Higher anxiety scores were found in participants with the most frequent intensive PA. In addition participants who had an intensive physical load less than once a week were at higher risk of anxiety and depression symptoms.

**TABLE 4:** Physical activity (PA), anxiety and depression.

**Characteristics of PA**		**Anxiety**		**Depression**	
		**Normal**	**Increased**	**Normal**	**Increased**
Type of PA at work, *n* (%)	sitting	1,129 (51.0)	1,083 (49.0)	1,594 (72.1)	618 (27.9)
	walking	676 (51.8)	628 (48.2)	915 (70.2)	389 (29.8)
	carrying loads	166 (47.0)	187 (53.0)	249 (70.5)	104 (29.5)
	heavy physical work	63 (45.0)	77 (55.0)	97 (69.3)	43 (30.7)
Chi-square/p-level		4.51/0.21		1.80/0.61	
Frequency of high PA, *n* (%)	<1 time a week	343 (46.7)	392 (53.3)	500 (68.0)	235 (32.0)
	1–2 times a week	575 (54.8)	475 (45.2)	822 (78.3)	228 (21.7)
	3–5 times a week	391 (53.4)	341 (46.6)	564 (77.0)	168 (23.0)
	>5 times a week	102 (44.3)	128 (55.7)	179 (77.8)	51 (22.2)
Chi-square/p-level		17.11/0.001		27.81/ < 0.0001	
Mean leisure-time walking, min., M ± m		73.1 ± 1.1	72.7 ± 1.1	73.5 ± 0.9	71.3 ± 1.4
t/p-level		0.26/0.79		1.28/0.20	

*Additionally individuals with increased depression scores more frequently than participants with normal depression scores had leisure-time walking less than 1 h a day [571 (46.5%) vs. 1,198 (41.9%), Chi-square = 7.4, and *p* = 0.006].*

### Sleep Quality, Anxiety, and Depression

All sleep problems analyzed in this study were tightly linked to anxiety and depression level (Table 5). Participants with sleep duration less than 6 h, with frequent difficulties in initiating and maintaining sleep, with daytime sleepiness and experience of sleep medication use have a significantly higher risk of having increased anxiety and depression scores in HADS.

**TABLE 5:** Sleep characteristics, anxiety, and depression.

**Sleep characteristics**		**Anxiety**		**Chi-square/p-level**	**Depression**		**Chi-square/p-level**
		**Normal**	**Increased**		**Normal**	**Increased**	
Mean sleep duration during last month, *n* (%)	<6 h	82 (37.4)	137 (62.6)	12.18/ < 0.001	121 (55.3)	98 (44.7)	22.49/ < 0.0001
	≥6 h	2,268 (49.5)	2,313 (50.5)		3,222 (70.3)	1,359 (29.7)	
Difficulty falling asleep, *n* (%)	≥3 times a week	200 (30.3)	459 (69.7)	105.86/ < 0.0001	393 (59.6)	266 (40.4)	36.21/ < 0.0001
	no or <3 times a week	2,150 (51.9)	1,991 (48.1)		2,950 (71.2)	1,191 (28.8)	
Difficulty staying asleep, *n* (%)	≥3 times a week	147 (29.1)	358 (70.9)	88.98/ < 0.0001	274 (54.3)	231 (45.7)	63.22/ < 0.0001
	no or <3 times a week	2,203 (51.3)	2,092 (48.7)		3,069 (71.5)	1,226 (28.5)	
Sleepiness: episodes of daytime falling asleep, *n* (%)	≥3 times a week	71 (31.7)	153 (68.3)	28.02/ < 0.0001	137 (61.2)	87 (38.8)	8.0/0.005
	no or <3 times a week	2,279 (49.8)	2,297 (50.2)		3,206 (70.1)	1,370 (29.9)	
Sleep medication use, *n* (%)	at least once	224 (29.4)	539 (70.6)	139.47/ < 0.0001	435 (57.0)	328 (43.0)	68.50/ < 0.0001
	never	2,126 (52.7)	1,911 (47.3)		2,908 (72.0)	1,129 (28.0)	

## Discussion

The present study was aimed at elucidating the link between different levels and types of PA and sleep. We found that having a high physical load six or more times a week is a risk factor for having insomnia symptoms, in particularly, sleep-onset difficulties.

High and regular PA is traditionally considered an important factor for physical and psychological well-being and an essential component of healthy lifestyle (Penedo and Dahn, 2005; Samitz et al., 2011; Cunningham et al., 2020). The role of exercise for improving quality of sleep, especially in persons with sleep problems, was also documented in a number of studies (Banno et al., 2018). Nevertheless, there are some controversies concerning the effects of different types of PA, its regimen, and intensity (Soltani et al., 2012; Wennman et al., 2014; Skarpsno et al., 2018a; Cillekens et al., 2020). The results of our study are in agreement with findings that physically demanding work and a very frequent intensive PA may have a negative impact on sleep quality, especially in terms of sleep initiating.

A number of social, psychological and physical variables may be proposed as mediators of the relationship between high physical load at work and sleep problems including musculoskeletal pain, physical exhaustion, social stress, social inequalities in physical and mental health, poor work hygiene, occupational stress and additional occupational hazards. These factors along with detailed information on physical well-being and chronic conditions should be taken into account in further research.

Our results indicate that the impact of very intensive PA on sleep quality is not limited to working conditions. We found that participants who practice very frequent (6 days a week or everyday) intensive PA are at higher risk of problems in initiating sleep irrespective of sociodemographic confounders. For adequate explanation this finding needs further investigation in terms of high PA, its causes and motivations. However, in general these results are in concordance with the evidence that vigorous PA may not have such benefits as moderate PA (Myllymäki et al., 2011; Pengpid and Peltzer, 2018) and a very high regular PA is linked to insomnia (Hartescu and Morgan, 2019). Thus, a very intensive PA may have a detrimental effect on sleep. One of possible mechanisms underlying this effect is hyperarousal state that represents the main feature of insomnia (Riemann et al., 2010). On the other hand, there could be a more complex relationship, when sleep problems and very high PA are determined by a third factor, e.g., body dissatisfaction or some difficult life circumstances.

The association between PA and sleep problems in our study could not be fully explained by emotional factors such as anxiety and depression, because no significant association between PA and psychological well-being was identified. On the other hand, as expected (Baglioni et al., 2011; Krystal, 2012), a tight link between sleep complaints, anxiety, and depression scores was found.

Contrary to our expectations we did not find any significant association between leisure-time walking and sleep quality when controlling for sociodemographic factors. This may be attributable to a relatively small percentage of participants with very low leisure-time walking and the role of some additional factors that were not analyzed in this study, such as the availability of personal vehicle.

### Limitations

Indeed, cross-sectional design of the study prevents definite conclusions about causal relationships between PA, sleep, anxiety and depression. As the study was screening and multidimensional, including a wide range of variables (Scientific Organizing Committee of the ESSE-RF…, 2013), no full standard questionnaires on sleep and PA were applied. We did not include in analysis compete data on chronic conditions and health behaviors that could be possible mediating variables and covariates.

In general the study emphasizes the necessity to consider the association between PA, sleep and psychological well-being as complex relationship. We found that excessively high PA is detrimental for sleep quality and poor sleep is linked to a wide range of emotional problems. Further research is needed to determine the role of social, psychosocial and physiological factors mediating these relationships as well as mechanisms underlying this association.

## Data Availability Statement

The raw data supporting the conclusions of this article will be made available by the authors, without undue reservation.

## Ethics Statement

The studies involving human participants were reviewed and approved by Local Ethical Committee (Almazov National Medical Research Centre), approval #193 dated by 8 October 2012. The patients/participants provided their written informed consent to participate in this study.

## Author Contributions

VA and ED: manuscript design, drafting manuscript, analysis and interpretation of data, and revising manuscript critically for important content. LK: manuscript design, drafting manuscript, analysis and interpretation of data, revising manuscript critically for important content, and approval for publication. OR: study concept and design, data collection and storage, data analysis, interpretation of data, revising manuscript critically for important content, and approval for publication. MaB: data collection and storage, data analysis, interpretation of data, and revising manuscript critically for important content. TS and MiB: interpretation of data and revising manuscript critically for important content. EB, RL, and DD: study concept and design, data collection and storage, data analysis, and interpretation of data. YS: manuscript design, drafting manuscript, analysis and interpretation of data, revising manuscript critically for important content, approval for publication, and accountable in ensuring that work-related questions are appropriately investigated/resolved. ES and AK: principal co-investigator, study concept and design, and approval for publication. All authors contributed to the article and approved the submitted version.

## Conflict of Interest

The authors declare that the research was conducted in the absence of any commercial or financial relationships that could be construed as a potential conflict of interest.

## Publisher’s Note

All claims expressed in this article are solely those of the authors and do not necessarily represent those of their affiliated organizations, or those of the publisher, the editors and the reviewers. Any product that may be evaluated in this article, or claim that may be made by its manufacturer, is not guaranteed or endorsed by the publisher.

## References

[B1] Baglioni C. Battagliese G. Feige B. Spiegelhalder K. Nissen C. Voderholzer U. (2011). Insomnia as a predictor of depression: a meta-analytic evaluation of longitudinal epidemiological studies. *J. Affect. Disord.* 135 10–19. 10.1016/j.jad.2011.01.011 21300408

[B2] Balanova I. U. A. Kontsevaia A. V. Shalnova S. A. Deev A. D. Artamonova V. G. Gatagonova T. M. (2014). Prevalence of behavioral risk factors for cardiovascular disease in the Russian population: results of the ESSE-RF epidemiological study. *Profil. Med.* 17 42–52.

[B3] Banno M. Harada Y. Taniguchi M. Tobita R. Tsujimoto H. Tsujimoto Y. (2018). Exercise can improve sleep quality: a systematic review and meta-analysis. *PeerJ* 6:e5172. 10.7717/peerj.5172 30018855 PMC6045928

[B4] Boitsov S. A Chazov E. I Shlyakhto E. V Shalnova S. A Konradi A. O Karpov Y. A (2013). Epidemiology of cardiovascular diseases in different regions of Russia (ESSE-RF). The rationale for and design of the study. *Prevent. Med.* 6 25–34.

[B5] Cillekens B. Lang M. van Mechelen W. Verhagen E. Huysmans M. A. Holtermann A. (2020). How does occupational physical activity influence health? An umbrella review of 23 health outcomes across 158 observational studies. *Br. J. Sports Med.* 54 1474–1481. 10.1136/bjsports-2020-102587 33239353

[B6] Cunningham C. O’ Sullivan R. Caserotti P. Tully M. A. (2020). Consequences of physical inactivity in older adults: a systematic review of reviews and meta-analyses. *Scand. J. Med. Sci. Sports.* 30 816–827. 10.1111/sms.13616 32020713

[B7] Dolezal B. A. Neufeld E. V. Boland D. M. Martin J. L. Cooper C. B. (2017). Interrelationship between Sleep and Exercise: a Systematic Review. *Adv. prevent. Med.* 2017:1364387. 10.1155/2017/1364387 28458924 PMC5385214

[B8] Ford D. E. Kamerow D. B. (1989). Epidemiologic study of sleep disturbances and psychiatric disorders. An opportunity for prevention?. *JAMA* 262 1479–1484. 2769898 10.1001/jama.262.11.1479

[B9] Hartescu I. Morgan K. (2019). Regular physical activity and insomnia: an international perspective. *J. Sleep Res.* 28:e12745. 10.1111/jsr.12745 30117220

[B10] Hartescu I. Morgan K. Stevinson C. D. (2015). Increased PA improves sleep and mood outcomes in inactive people with insomnia: a randomized controlled trial. *J. Sleep Res.* 24 526–534. 10.1111/jsr.12297 25903450

[B11] King A. C. Oman R. F. Brassington G. S. Bliwise D. L. Haskell W. L. (1997). Moderate-intensity exercise and self-rated quality of sleep in older adults: a randomized controlled trial. *JAMA* 277 32–37. 8980207

[B12] King A. C. Oman R. F. Brassington G. S. Bliwise D. L. Haskell W. L. (2008). Effects of moderate-intensity exercise on polysomnographic and subjective sleep quality in older adults with mild to moderate sleep complaints. *J. Gerontol. A Biol. Sci. Med. Sci.* 63 997–1004. 18840807 10.1093/gerona/63.9.997 PMC7182081

[B13] Kline C. E. (2014). The bidirectional relationship between exercise and sleep: implications for exercise adherence and sleep improvement. *Am. J. lifestyle Med.* 8 375–379. 10.1177/1559827614544437 25729341 PMC4341978

[B14] Korostovtseva L. S. Alieva A. S. Rotar O. P. (2020). Sleep duration, lipid profile and insulin resistance: potential role of lipoprotein(a). *Int. J. Mol. Sci.* 21:4680. 10.3390/ijms21134680 32630105 PMC7369827

[B15] Krystal A. D. (2012). Psychiatric disorders and sleep. *Neurol. Clin.* 30 1389–1413. 23099143 10.1016/j.ncl.2012.08.018 PMC3493205

[B16] Myllymäki T. Kyröläinen H. Savolainen K. Hokka L. Jakonen R. Juuti T. (2011). Effects of vigorous late-night exercise on sleep quality and cardiac autonomic activity. *J. Sleep Res.* 20 146–153. 20673290 10.1111/j.1365-2869.2010.00874.x

[B17] Penedo F. J. Dahn J. R. (2005). Exercise and well-being: a review of mental and physical health benefits associated with PA. *Curr. Opin. Psychiatry* 18 189–193. 10.1097/00001504-200503000-00013 16639173

[B18] Pengpid S. Peltzer K. (2018). Vigorous PA, perceived stress, sleep and mental health among university students from 23 low- and middle-income countries. *Int. J. Adolesc. Med. Health* 32:2020. 10.1515/ijamh-2017-0116 29331097

[B19] Reid K. J. Baron K. G. Lu B. Naylor E. Wolfe L. Zee P. C. (2010). Aerobic exercise improves self-reported sleep and quality of life in older adults with insomnia. *Sleep Med.* 11 934–940. 20813580 10.1016/j.sleep.2010.04.014 PMC2992829

[B20] Riemann D. Baglioni C. Bassetti C. Bjorvatn B. Dolenc Groselj L. Ellis J. G. (2017). European guideline for the diagnosis and treatment of insomnia. *J Sleep Res.* 26 675–700. 10.1111/jsr.12594 28875581

[B21] Riemann D. Spiegelhalder K. Feige B. Voderholzer U. Berger M. Perlis M. (2010). The hyperarousal model of insomnia: a review of the concept and its evidence. *Sleep Med. Rev.* 14 19–31. 10.1016/j.smrv.2009.04.002 19481481

[B22] Samitz G. Egger M. Zwahlen M. (2011). Domains of PA and all-cause mortality: systematic review and dose—response metaanalysis of cohort studies. *Intern. J. Epidemiol.* 40 1382–1400. 10.1093/ije/dyr112 22039197

[B23] Sivertsen B. Madsen I. E. Salo P. Tell G. S. Øverland S. (2015). Use of Sleep Medications and Mortality: the Hordaland Health Study. *Drugs Real World Outcomes* 2 123–128. 10.1007/s40801-015-0023-8 27747767 PMC4883191

[B24] Skarpsno E. S. Mork P. J. Nilsen T. I. L. Jørgensen M. B. Holtermann A. (2018a). Objectively measured occupational and leisure-time physical activity: cross-sectional associations with sleep problems. *Scand. J. Work Environ. Health* 44 202–211. 10.5271/sjweh.3688 29094169

[B25] Skarpsno E. S. Nilsen T. I. L. Sand T. Hagen K. Mork P. J. (2018b). Physical work exposure, chronic musculoskeletal pain and risk of insomnia: longitudinal data from the HUNT study. *Norway. Occup. Environ. Med.* 75 421–426. 10.1136/oemed-2018-105050 29674486

[B26] Smulevich A. B. (2007). *Depressions in General Practice.* Moscow: Meditsina Publishers.

[B27] Soltani M. Haytabakhsh M. R. Najman J. M. Williams G. M. O’Callaghan M. J. Bor W. (2012). Sleepless nights: the effect of socioeconomic status, PA, and lifestyle factors on sleep quality in a large cohort of Australian women. *Arch. Womens Ment. Health* 15 237–247. 10.1007/s00737-012-0281-3 22585289

[B28] Uchida S. Shioda K. Morita Y. Kubota C. Ganeko M. Takeda N. (2012). Exercise effects on sleep physiology. *Front. Neurol.* 3:48. 10.3389/fneur.2012.00048 22485106 PMC3317043

[B29] Vancampfort D. Stubbs B. Smith L. Hallgren M. Firth J. Herring M. P. (2018). and sleep problems in 38 low- and middle-income countries. *Sleep Med.* 48 140–147. 10.1016/j.sleep.2018.04.013 29908426

[B30] Vanderlinden J. Boen F. van Uffelen J. (2020). Effects of PA programs on sleep outcomes in older adults: a systematic review. *Int. J. Behav. Nutr. Phys. Act.* 17:11. 10.1186/s12966-020-0913-3 32024532 PMC7003368

[B31] Watson N. F. Badr M. S. Belenky G. Bliwise D. L. Buxton O. M. Buysse D. (2015). Recommended Amount of Sleep for a Healthy Adult: a Joint Consensus Statement of the American Academy of Sleep Medicine and Sleep Research Society. *Sleep* 38 843–844. 10.5665/sleep.4716 26039963 PMC4434546

[B32] Wennman H. Kronholm E. Partonen T. Tolvanen A. Peltonen M. Vasankari T. (2014). PA and sleep profiles in Finnish men and women. *BMC Public Health* 14:82. 10.1186/1471-2458-14-82 24467881 PMC3914355

[B33] World Health Organisation (WHO) (2010). Global Recommendations on PA for Health. Geneva: World Health Organization.

[B34] Yang P. Y. Ho K. H. Chen H. C. Chien M. Y. (2012). Exercise training improves sleep quality in middle-aged and older adults with sleep problems: a systematic review. *J. Physiother.* 58 157–163. 10.1016/S1836-9553(12)70106-6 22884182

[B35] Zigmond A. S. Snaith R. P. (1983). The Hospital Anxiety and Depression Scale. *Acta Psychiatr. Scand.* 67 361–370. 10.1111/j.1600-0447.1983.tb09716.x 6880820

