# Supplementary material for: Physical Activity Is Associated With Sleep Quality: Results of the ESSE-RF Epidemiological Study
Source: Front Psychol. 2021 Aug 5;12:705212. doi: 10.3389/fpsyg.2021.705212 (PMC8383779; doi:10.3389/fpsyg.2021.705212)
Supplement: Supplementary file 1 [file Table_1.DOCX]

| Question | Answer options |
| --- | --- |
| **Sleep duration** |  |
| How long have you been sleeping daily during the last month? | Numerical answer (in hours) |
| **Sleep-related complaints** |  |
| How often did you have difficulties in falling asleep for ≥30 min after going to bed during the last month? | Never  < 1 time per week  1–2 times per week  ≥3 times per week |
| How often did you have difficulties in falling asleep after midnight awakening during the last month? | Never  < 1 time per week  1–2 times per week  ≥3 times per week |
| How often have you had difficulties in staying awake when required? | Never  < 1 time per week  1–2 times per week  ≥3 times per week |
| **Sleeping pills intake** |  |
| How often have you taken sleeping pills during the last month? | Never  < 1 time per week  1–2 times per week  ≥3 times per week |
| **Physical activity** |  |
| Which type of physical activity do you usually perform at work? | Mainly sitting  Mainly walking  Lifting and carrying small loads  Heavy physical work |
| How many times per week do you perform heavy physical load lasting for at least 20-30 minutes at the level that you develop mild dyspnea or sweating? | Never  < 1 time per week  1–2 times per week  ≥3 times per week |
| How long do you spend walking per day, including the way to and back from your workplace? | Numerical answer (in minutes) |

Supplemental Table 1

List of questions from the survey used for the analysis of sleep quality and physical activity
